# Should we abandon regional anesthesia in open inguinal hernia repair in adults?

**DOI:** 10.1186/s40001-015-0170-0

**Published:** 2015-09-17

**Authors:** B. Bakota, M. Kopljar, S. Baranovic, M. Miletic, M. Marinovic, D. Vidovic

**Affiliations:** Department of Surgery, General Hospital Karlovac, Karlovac, Croatia; Department of Surgery, Clinical Hospital Dubrava, Av. Gojka Suska 6, 10000 Zagreb, Croatia; Department of Anesthesiology and Intensive Care Unit, University Hospital Center “Sestre Milosrdnice”, Zagreb, Croatia; Department of Surgery, University Hospital Center Rijeka, Rijeka, Croatia; Department of Surgery, University Hospital Center “Sestre Milosrdnice”, Zagreb, Croatia

**Keywords:** Hernia, Meta-analysis, Systematic review, Regional anesthesia, General anesthesia, Complications

## Abstract

Inguinal hernia repair is a common worldwide surgical procedure usually done in the outpatient setting. The purpose of this systematic review is to make an evidence-based meta-analysis to determine the possible benefits of regional (neuraxial block) anesthesia compared to general anesthesia in open inguinal hernia repair in adults. Cochrane Library, Medline, EMBASE, CINAHL, SCI-EXPANDED, SCOPUS as well as trial registries, conference proceedings and reference lists were searched. Only randomized controlled trials (RCT) that compare neuraxial block (spinal or/and epidural) anesthesia (NABA) and general anesthesia (GA) were included. Main outcome measures were postoperative complications, urinary retention and postoperative pain. Seven RCTs were included in this review. A total of 308 patients were analyzed with 154 patients in each group. Overall complications were evenly distributed in NABA and in GA group [OR 1.17, 95 % CI (0.52–2.66)]. Urinary retention was statistically less frequent in GA group compared to NABA group [OR 0.25, 95 % CI (0.08–0.74)]. Movement-associated pain score 24 h after surgery was significantly lower in NABA group [SMD 5.59, 95 % CI (3.69–7.50)]. Time of first analgesia application was shorter in GA group [SMD 8.99, 95 % CI 6.10–11.89]. Compared to GA, NABA appears to be a more adequate technique in terms of postoperative pain control. However, when GA is applied, patients seem to have less voiding problems.

## Background

Inguinal hernia repair is one of the most common procedures in general surgery performed with the estimation of a 20 million surgeries per year [1]. Local (LA), regional (RA) or general anesthesia (GA) enable the variety of surgical procedures for open inguinal hernia in adults, in which, according to the data from Scotland [2], Sweden [3] and Danish Hernia Database collaboration [4, 5], GA has a frequency of 60-70 %, RA 10-20 % and LA 10 %. In spite of current Danish Hernia Database recommendations that RA (spinal or epidural) should be abandoned [6], it is still used in 10-20 % of procedures [1, 7]. Although the current literature does not favor the use of RA, there are still no clear guidelines/evidence-based proof to abandon it. The purpose of this systematic review is to make evidence-based analysis in order to determine the possible benefits of regional (neuraxial block) anesthesia (NABA) in open inguinal hernia repair in adults. Within this meta-analysis, we compared NABA with GA as the most frequent type of anesthesia used in open inguinal hernia repair in adults [1, 5, 8].

## Review

We applied the methods according to Cochrane Collaboration standards [9] and to the protocol published [10]. The inclusion criteria were randomized controlled trials (RCT) only, that compare neuraxial (spinal and/or epidural) block anesthesia (NABA) with general anesthesia (GA) for open inguinal hernia repair in adults, irrespective of the language reported on. All the patients with a clinical diagnosis of inguinal hernia, which involves primary inguinal hernia, unilateral, bilateral or recurrent hernia that had an indication for an appropriate surgical management, were included.

Publications with repeated results together with double publications were excluded from this study. Studies that included a double anesthetic procedure to the same group of patients were also excluded.

We defined complications, urinary retention and postoperative pain as the main outcome measures.

Complications: Major complications included significant respiratory and circulatory complications (hyper/hypotension) as well as other potentially life-threatening visceral and vascular injuries. Minor complications were defined as the ones which do not require an additional hospital treatment (surgical site infection, hematoma, headache, nausea/vomiting, sore throat, conversion, etc.). Hematoma includes seroma and a wound hematoma. Conversion defines an alteration of anesthesia type (from neuraxial to general).

Urinary retention was defined as a need for catheterization due to lack of micturition.

Postoperative pain was defined as groin, thigh or testicular pain at a time point measured after the operation with a need for postoperative analgesia; it was evaluated through the need for postoperative analgesia, duration of postoperative analgesia, movement-associated pain score and the time of first request for analgesic. Length of hospital stay was addressed in time units noted. Time to ambulation was defined as a time from the end of surgery to a moment when the patient was able to stand and walk with crutches. Time to full mobility was defined as a time from the end of surgery to a moment when the patient was able to stand and walk without assistance. Return to work defines the time, measured in days, from surgery to ordinary working activities. Patient satisfaction is a major component used for measuring the quality of health care.

We searched the Cochrane Library, MEDLINE, EMBASE, CINAHL, SCI-EXPANDED, SCOPUS as well as trial registries, conference proceedings and reference lists. We identified the trials up to September 2014. Methodological quality for all the studies was assessed in accordance with the Cochrane Collaboration guidelines by two independent reviewers. If opinion diversity existed, other review team members arbitrated.

Assessment of risk of bias in included studies was done using The Cochrane Collaboration’s risk of bias tool as described in Chapter 8 of the Cochrane Handbook for Systematic Reviews of Interventions [9].

The review authors were not blinded to the names of the authors, institutions, journal or results of a study.

The data were gathered into the electronic spreadsheet, and statistical analysis with RevMan 5.3 was performed. Dichotomous outcomes data were analyzed with Mantel–Haenszel odds ratio (OR) method, whilst for continuous outcome data, the weighted standardized mean difference (SMD) was used. The results were calculated and reported using DerSimonian–Laird random-effect model (RE). Aside from meta-analysis, if sufficient number of studies showed the data for the same outcome, a Number Needed to Treat (NNT) calculation [11] was performed.

## Results

**Fig. 1:**
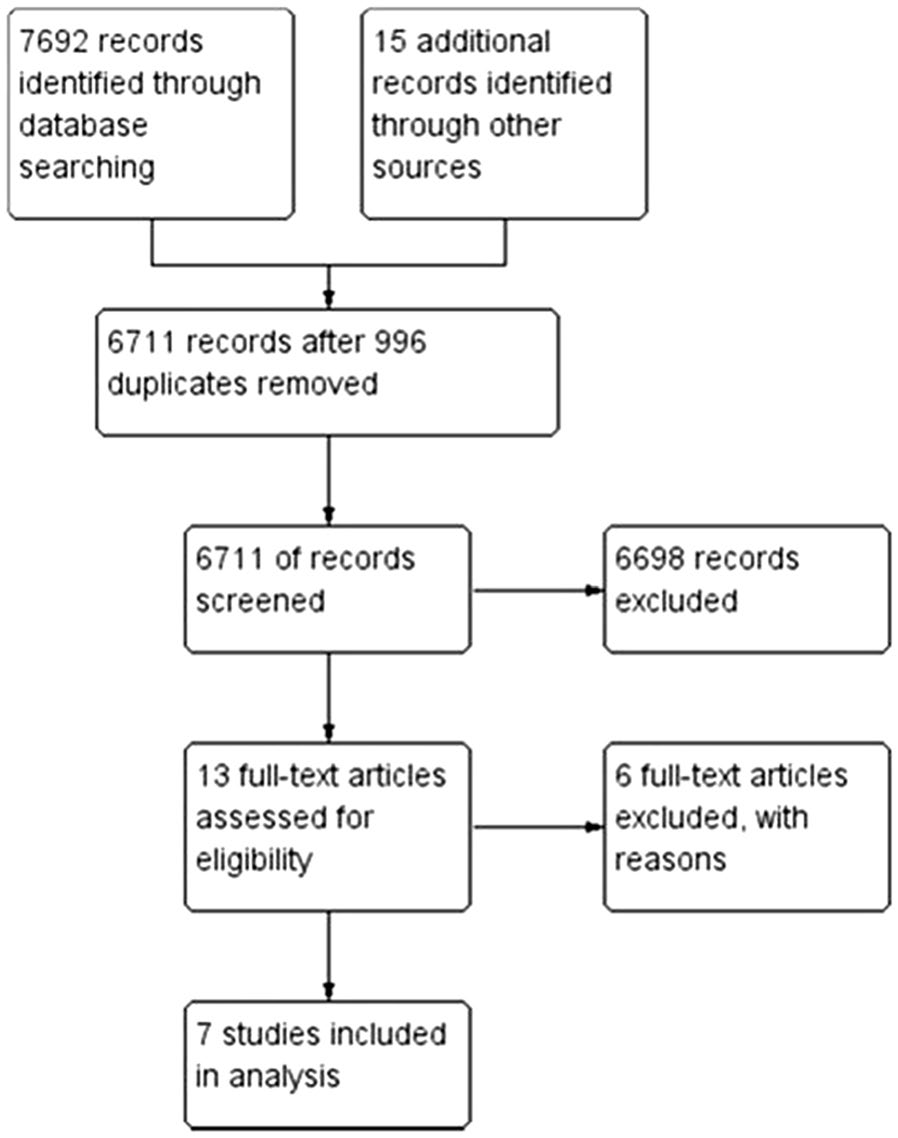
PRISMA flow diagram describing the article search and inclusion in meta-analysis

The initial search of electronic databases gave us a result of 7692 studies. We also identified 15 additional articles through reading the references of previously mentioned studies. After we have eliminated the studies repeated in different databases, a 6711 potentially relevant articles for further analysis remained. We identified 13 studies that could not be excluded based solely on title and abstract. With further analysis of these studies, we eliminated additional six studies. The remaining seven studies matched the above determined criteria for this meta-analysis. This is described in PRISMA flow diagram (Fig. 1).

### Characteristics of included studies

A total of 308 patients from seven RCTs (Burney [8]; Varshney [12]; Godfrey [13]; Merhav [14]; Ozgün [15]; Srivastava [16]; Tverskoy [17]) were analyzed with 154 patients in each group.

All of the included studies were published in English. Five of the studies [12–16] had three groups that compared LA, GA and RA. From these studies, we used the data for NABA and GA only. The remaining two studies [8, 17] applied the main design of our meta-analysis.

### Characteristics of excluded studies

We excluded six trials from our research for the following reasons: two of the studies were not randomized [18, 19], one study was a meta-analysis [20], one study was repeated [3] and in two studies the two different types of anesthesia were simultaneously applied [21, 22].

### Risk of bias in included studies

All included studies provided information on design and methodology. The authors’ evaluation for risk of bias of each study is shown in Table 1.

**Table 1:** Risk of bias summary: review authors’ judgements about each risk of bias item for each included study

Publication year	Author	Randomization	Allocation	Blinding of patients and medical personnel	Blinding of outcome assessment	Incomplete outcome data	Selective reporting	Other bias
1981	Godfrey	1	1	1	1	3	3	2
1990	Tverskoy	3	1	3	1	3	3	3
1993	Merhav	3	3	1	1	1	3	2
2002	Ozgun	3	1	1	1	3	3	3
2004	Burney	3	3	1	1	1	3	3
2007	Srivastava	1	1	1	1	3	3	3
2009	Varshney	1	1	1	1	1	3	3

1 = high risk, 2 = unclear risk, 3 = low risk

Randomization (sequence generation) was mentioned in all included studies. Adequate sequence generation was clearly described in four of the studies [8, 14, 15, 17]. Although randomization was mentioned, the method of sequence generation was not described in three articles [12, 13, 16].

Allocation sequence was adequately concealed in two studies [8, 14], while in the remaining five studies there was no report of any attempt to conceal the allocation sequence.

Blinding of patients and medical personnel was reported in only one study [17], while blinding of outcome assessment was not mentioned in any of the studies, so they might involve a high risk of bias.

Four studies [13, 15–17] were judged to have a low risk of bias in relation to incomplete outcome data. In two studies [8, 12], no standard deviations were presented for length of stay. In one study [14], eleven patients were omitted postoperatively from the study without reported reasons.

All of the seven studies were judged to have a low risk of selective outcome reporting.

Risk of other potential sources of bias was judged as low in five studies. One of the studies was conducted in 1981 [13] before the awareness of conflict of interest issues became more widespread [23], yet still clearly disclosed the source of financial support. Two of the studies [13, 14] did not present the ethical committee approval and one study was without the data of patient consent [13]. In only one study [15] hernia classification was done, whilst in three [13, 15, 17] the technique of repair was noted.

### Outcomes

**Fig. 2:**
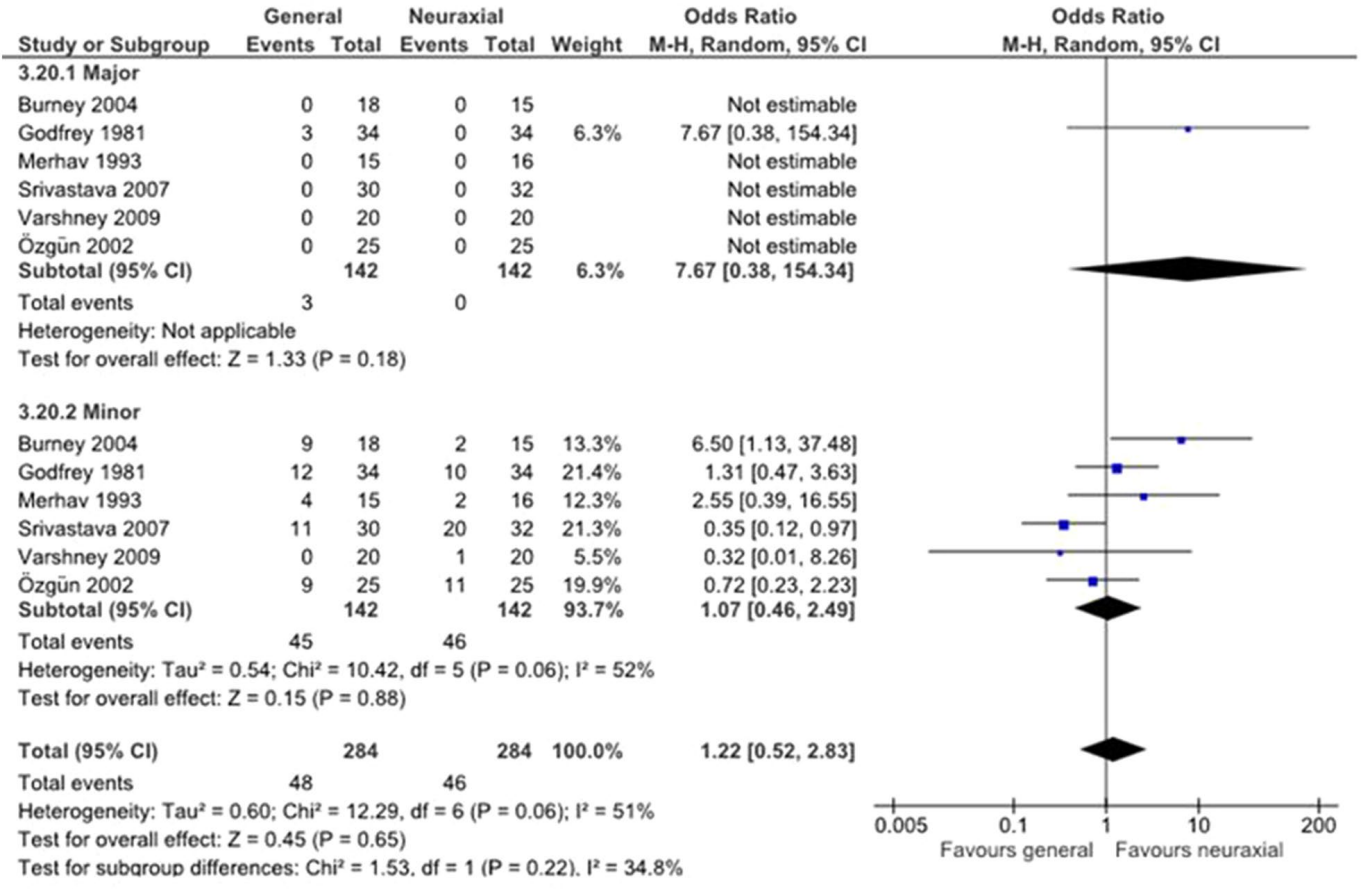
The incidence of major and minor complications

Only one study [8, 13] reported of three patients operated under general anesthesia that had major complications (Fig. 2), with no statistically significant difference (OR 7.67, 95 % CI 0.038–154.34). NNT was calculated; in order to prevent one major complication it is necessary to operate 47.33 patients in NABA instead of under GA.

Six studies [8, 12–16] reported on minor complications: 46 patients in NABA group and 45 in GA group, but also without statistically significant difference (OR 1.07, 95 % CI 0.46–2.49) (Fig. 2). An NNT calculation informs us that in order to avoid one minor complication 142 patients should undergo GA instead of NABA.

**Fig. 3:**
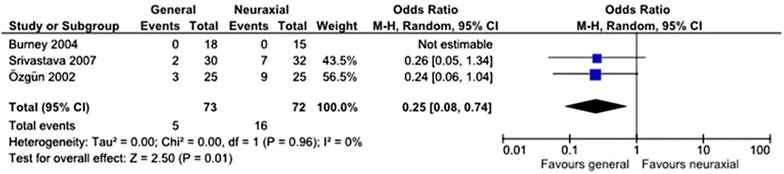
The incidence of urinary retention when GA and NABA are compared

Three studies [8, 15, 16] assessed urinary retention and the OR was 0.25 (95 % CI 0.08–0.74) in favor of general anesthesia with no evidence of heterogeneity (*I*^2^ = 0 %; *p* = 0.96) (Fig. 3).

Sore throat reported in one study was expectedly more frequent in patients treated in GA (OR 8.27, 95 % CI 0.41–167.23) [16] with no statistical significance. Nausea and vomiting reported in four studies [8, 12, 15, 16] were more frequent after general anesthesia (12 against 6 cases), also without statistically significant difference (OR 2.08, 95 % CI 0.73–5.98). One study [13] reported of circulatory problems that had a higher occurrence rate in patients treated in NABA, also with no statistically significant difference (OR 0.32, 95 % CI 0.01–8.23). Two studies [8, 16] that assessed headache reported its higher occurrence rate in patients treated in NABA (five against two cases), without statistical significance (OR 0.39, 95 % CI 0.07–2.27). One study [13] also reported on postoperative cough problems which were more frequent in patients treated in GA, also without statistical significance (OR 1.55, 95 % CI 0.24–9.91). Surgical site infections described in two articles [13, 15] were more frequent in patients treated in GA with no statistical significance (OR 1.39, 95 % CI 0.45–4.29). Hematoma reported in three studies [13, 15, 16] was more often in patients treated in GA (nine against seven cases) also with no statistical difference (OR 1.39, 95 % CI 0.45–4.29). No neurological complications were reported.

One study [8] reported on conversions from NABA to GA. Altogether, only one patient (6.67 %) was converted which showed no statistical significance (OR 0.26; 95 % CI 0.01–6.90). According to the NNT calculation, in order to avoid one conversion 142 patients should be operated in GA instead in NABA.

Two studies [8, 15] reported on shorter length of stay when patients were anesthetized in neuraxial block (SMD 0.44, 95 % CI -0.12 to 1.00), but without statistical significance.

### Postoperative pain

One study [16] reported on the duration of postoperative analgesia and found that the SMD was -1.41 (95 % CI -1.97 to 0.85) in favor of spinal anesthesia.

One study [17] reported on movement-associated pain score 24 h after procedure and according to 1-100 Visual Analogue Scale (VAS), the findings were in favor of NABA with statistical significance (SMD 5.59, 95 % CI 3.69–7.50).

Two studies [8, 13] reported on number of patients that had a need for postoperative analgesia. Patients treated in GA had a higher need of postoperative analgesia than the ones treated in NABA (47 against 37), with no statistical significance (OR 1.56, 95 % CI 0.60–4.04).

One study [17] reported the time from the end of surgery to the first request for analgesic and found that SMD was 8.99 (95 % CI 6.10–11.89) in favor of NABA which was statistically significant.

Time to ambulation 6 h after surgery was reported in one study [16] and was almost equal in patients operated under NABA and GA, without statistical significance (OR 0.02, 95 % CI -0.17 to 0.21).

One study [15] reported the time to full mobility and found that SMD was -0.50 (95 % CI -3.44 to 2.44) in favor of GA which was not statistically significant.

Two studies [13, 15] reported on a faster return to work after GA, but without statistical significance (SMD -0.14, 95 % CI -0.51 to 0.22).

Four studies [8, 12, 15, 16] reported on patient satisfaction (79 against 76 events) in favor of patients treated in GA, without significant statistical difference (OR 1.10, 95 % CI 0.45–2.67).

## Discussion

Overall, the quality of clinical trials within this meta-analysis in terms of design, reporting and methodology were acceptable. Nevertheless, insufficient quality of reporting in some of the included studies resulted in substantial uncertainties in the risk of bias assessment. For example, only two of the included studies clearly demonstrated both adequate sequence generation and concealment of the sequence allocation [8, 14] (Table 1).

In a summary of risk of bias for each study across domains, three studies were considered to have a high risk of bias [12, 13, 16], another three to be with an unclear risk of bias [8, 14, 15], and one to have a low risk of bias [17].

The key study-level domains were randomization, allocation and completeness of outcome data. Blinding of participants was obviously not possible in most cases, but as the outcomes assessed in this study are mostly not dependent on patient’s knowledge of the anesthetic procedure this was regarded as low risk. Blinding of outcome assessment was reported in neither of the studies. Although it was probably performed, it was still considered high risk. Selective reporting risk was low due to the fact that all of the outcomes were reported as stated in respective methods sections.

Some of the outcomes in studies included in this meta-analysis were heterogeneous and some of the studies had a small sample size, thus decreasing the quality of evidence. Only one study did not report minor complications [17] and its main focus was postoperative pain.

Surgical technique (tension/tension free) as well as hernia classification type were neither primarily analyzed nor discussed in relation to the study outcomes within most of the studies, and the insufficiency of this data could increase the risk of bias especially due to the fact that some studies neither mentioned the surgical technique nor have they mentioned the hernia classification type [8, 12, 16].

The results of this meta-analysis provide evidence that when NABA is applied in open inguinal hernia repair in adults, time to ambulation is shorter and pain is less present than in GA. This also stands for sore throat, cough, nausea/vomiting, surgical site infections, wound hematoma as well as for the length of hospital stay but without significant statistical benefit. However, one study [8] mentioned no standard deviation for the length of hospital stay, therefore there is a possibility that this outcome may be biased.

A rate of major complications also favors NABA in comparison with GA, but without statistical significance. On the other hand, when GA is applied there seems to be less urinary retentions. In GA, there also appears to be a lower rate of minor complications in respect to circulatory problems and headache, but without statistical significance. The same stands for time to full mobility, return to work and patient satisfaction.

### Agreements and disagreements with other studies or reviews

In terms of certain risks that refer mostly to cardio-respiratory comorbidities, the contemporary development of short-acting anesthetic drugs allow GA to be appropriate for a day surgery [8]. Still, particular complications related to GA such as nausea, vomiting, cough, headache and voiding problems which prolong hospital treatment may be present. On the other hand, NABA has the advantage of avoiding paralytic agents and endotracheal intubation, but has the disadvantage of being associated with slow recovery of sensory and motor function (depending on anesthetic type and dose), long recovery room time, as well as retention of urine [8]. Even though there is a consensus on the choice of surgical treatment, the one that is related to the type of anesthesia is still to be determined.

Although GA is still the most frequent choice of anesthesia [1], it is not suitable for all patients, especially when considering a relationship with certain complications such as, circulatory and respiratory problems, nausea and vomiting [24]. On the other hand, it is considered that regional anesthesia decreases the risks related to general anesthesia, provides a more adequate pain control after surgery and earlier patient dismission, therefore lowering the costs [21, 25]. In addition, it may present a more adequate alternative in patients with respiratory problems such as chronic obstructive pulmonary disease (COPD) [26, 27]. Nevertheless, the incidence of postoperative urinary retention following regional anesthesia is much higher compared to other anesthetic techniques [6, 28, 29]. Furthermore, a commonly recognized complication in regional anesthesia is post dural puncture headache after spinal anesthesia [30] or inadvertent dural puncture with epidural anesthesia. Although regional anesthesia may seem to be a reasonable alternative to general anesthesia in American Society of Anesthesiologists (ASA) grade 3 and 4 patients with cardiovascular comorbidity [31, 32], it still requires specialist anesthetic evaluation and monitoring as well as recovery facilities equal to when general anesthesia is administered [33]. Yet, general anesthesia remains a technique of choice for uncooperative or anxious patients, difficult repairs (reoperation after a mesh repair), and in situations when other anesthetic techniques fail to provide an adequate surgical condition [21].

 Overall, the studies involved in this meta-analysis have shown that the general complication rates between GA and NABA are very similar, although an NNT analysis for major complications is in a slight favor of NABA. On the other hand, the results of this meta-analysis point out that NABA can provide a more sufficient postoperative pain control compared to GA. Likewise, complications such as nausea and vomiting are less frequent when NABA is applied [8, 15, 16]. The results of a few studies have demonstrated that NABA has less adverse effects on respiratory function (cough, sore throat) in comparison with GA [13, 27]. Also, the application of NABA as well as GA shows similar incidences of wound infection as well as of postoperative hematoma. Still, the urinary retention rate favors the use of GA [15, 16, 28, 29].

## Conclusions

### Implications for practice

A direct comparison of NABA and GA has shown the differences of particular outcomes of these two anesthetic techniques.

Contrary to some experts that advocate regional anesthesia (NABA in particular) as a type of anesthesia that should be abandoned, the results of this meta-analysis indicate that there is indeed a place for NABA in open inguinal hernia repair in adults, especially in certain patients with ASA 3-4 with cardiovascular (and pulmonic) comorbidities. While GA compared to NABA is associated with lower frequency of urinary retentions, the use of NABA compared to GA results in a better postoperative pain control.

Overall, we should always have a guideline in our mind; besides the anesthesia–patient relation, as well as surgeon–patient relation, an interaction between the surgeon and the anesthetist may sometimes play an important role in deciding which type of anesthesia should be used in a particular case [8, 19].

### Implications for further research

In order to determine a more sufficient impact factor of complications such are respiratory and circulatory, as well as headache, nausea and vomiting, further well-structured RCTs of this sort should be performed to obtain a greater sample size.

## Abbreviations

RCT: randomized controlled trials
NABA: neuraxial block anesthesia
GA: general anesthesia
LA: local anesthesia
RA: regional anesthesia
OR: Mantel–Haenszel odds ratio
SMD: standardized mean difference
RE: DerSimonian–Laird random-effect model
NNT: number needed to treat
VAS: visual analogue scale
COPD: chronic obstructive pulmonary disease
ASA: American Society of Anesthesiologists
